# Characterization of SV-40 Tag rats as a model to study prostate cancer

**DOI:** 10.1186/1471-2407-9-30

**Published:** 2009-01-26

**Authors:** Curt E Harper, Brijesh B Patel, Leah M Cook, Jun Wang, Tomoyuki Shirai, Isam A Eltoum, Coral A Lamartiniere

**Affiliations:** 1 Department of Pharmacology and Toxicology, University of Alabama at Birmingham, Birmingham, Alabama, USA; 2 Department of Experimental Pathology and Tumor Biology, Nagoya City University Graduate School of Medical Sciences, Nagoya, Japan; 3 Department of Pathology, University of Alabama at Birmingham, Birmingham, Alabama, USA; 4 UAB Comprehensive Cancer Center, University of Alabama at Birmingham, Birmingham, Alabama, USA

## Abstract

**Background:**

Prostate cancer is the second most frequently diagnosed cancer in men. Animal models that closely mimic clinical disease in humans are invaluable tools in the fight against prostate cancer. Recently, a Simian Virus-40 T-antigen (SV-40 Tag) targeted probasin promoter rat model was developed. This model, however, has not been extensively characterized; hence we have investigated the ontogeny of prostate cancer and determined the role of sex steroid receptor and insulin-like growth factor-1 (IGF-1) signaling proteins in the novel SV-40 Tag rat.

**Methods:**

The SV-40 Tag rat was histopathologically characterized for time to tumor development, incidence and multiplicity and in the ventral, dorsal, lateral and anterior lobes of the prostate. Immunoassay techniques were employed to measure cell proliferation, apoptosis, and sex steroid receptor and growth factor signaling-related proteins. Steroid hormone concentrations were measured *via *coated well enzyme linked immunosorbent assay (ELISA) kits.

**Results:**

Prostatic intraepithelial neoplasia (PIN) and well-differentiated prostate cancer developed as early as 2 and 10 weeks of age, respectively in the ventral prostate (VP) followed by in the dorsolateral (DLP). At 8 weeks of age, testosterone and dihydrotestosterone (DHT) concentrations in SV-40 Tag rats were increased when compared to non-transgenic rats. High cell proliferation and apoptotic indices were found in VP and DLP of transgenic rats. Furthermore, we observed increased protein expression of androgen receptor, IGF-1, IGF-1 receptor, and extracellular signal-regulated kinases in the prostates of SV-40 Tag rats.

**Conclusion:**

The rapid development of PIN and prostate cancer in conjunction with the large prostate size makes the SV-40 Tag rat a useful model for studying prostate cancer. This study provides evidence of the role of sex steroid and growth factor proteins in prostate cancer development and defines appropriate windows of opportunity for preclinical trials and aids in the rational design of chemoprevention, intervention, regression, and therapeutic studies using prostate cancer rodent models.

## Background

Prostate cancer is the second most frequently diagnosed cancer in men, with 782,600 new cases projected to occur in 2007 [1]. It was estimated that 254,000 deaths would occur from prostate cancer in the past year. One of the first means of prostate cancer treatment was androgen deprivation and estrogen administration [2]. Hence, it is known that sex steroids and their receptors play a major role in prostate cancer etiology. The androgen receptor (AR) is believed to participate in prostate cancer progression, including its activation and up-regulation, point mutations, and ligand-independent activation. Testosterone, a ligand for the AR, is secreted primarily by the testes and is subsequently converted to DHT *via *the enzyme, 5-α-reductase. DHT has a 50-fold higher binding affinity for AR and is 10 times more potent than testosterone [3]. Whether or not elevated blood levels of androgens are a significant risk factor for prostate cancer is open for debate. Some have reported that increased testosterone levels in the blood are associated with an increased risk of prostate cancer [4]. Others have failed to support the "androgen hypothesis" that circulating testosterone and DHT are positively associated with prostate cancer risk [5].

Estrogens and their receptors, including estrogen receptor-alpha (ER-alpha) and estrogen receptor-beta (ER-beta), play an integral role in normal growth, differentiation, and development of the prostate. In a study of over 600 men, low circulating concentrations of estradiol were associated with a decreased risk of prostate cancer [4]. In the past, estrogens have been used to treat prostate cancer because it resulted in prostate growth inhibitory effects. However, toxicity and poor response rate associated with estrogen therapy have hindered its progress in treating prostate cancer. The timing of administration and dose often dictates the type of response produced by estrogens or estrogen-like chemicals [6].

Growth factor signaling also plays a critical role in the growth and development of the prostate, including the proteins of the insulin-like growth factor axis. The two growth factors, IGF-1 and IGF-2, interact with 6 known IGF-binding proteins (IGF-BPs), which regulate binding to the 2 IGF receptors (IGF-1R and IGF-2R). Elevated levels of IGF-1 and decreased levels of IGF-BP3 in the blood serum have been associated with an increased risk of advanced stage prostate cancer [7]. Additional support to the role of IGF-1 and prostate growth can be found in IGF-1 deficient mice that exhibit decreased prostate size [8] and increased rat prostate growth with systemic administration of IGF-1 [9]. The expression of IGF-1R in prostate cancer remains controversial. It has been proposed that reduced IGF-1R action is necessary for prostate cancer progression. Tennant *et al. *reported that IGF-1R expression is abundant in normal and early-stage tumors, but reduced in advanced and metastatic prostate cancer [10]. The IGF-1 signal transduction cascade ultimately leads to the phosphorylation of intracellular substrates and activation of the mitogen-activated protein kinase/extracellular signal-regulated kinase (MAPK/ERK) pathway. The MAPK/ERK pathway is involved in cell differentiation, cell survival, and cell migration [11]. ERKs, downstream effectors of growth factor and sex steroid receptor signaling, also regulate cell proliferation and apoptosis and participate in prostate carcinogenesis [11]. Furthermore, the MAPK/ERK signaling has the ability to re-activate the AR pathway by a hormone-independent mechanism that may lead to androgen-independent prostate cancer [12].

Animal models that closely mimic clinical disease in humans are invaluable tools in the fight against prostate cancer. The TRAMP model developed in 1995 has been used extensively as a prostate cancer model over the past decade [13]. One limitation of the TRAMP model, however, is that it is mouse-based; therefore, the prostates are very small and necessitate several dissected prostate lobes to allow extensive mechanism of action research at the protein level. Recently, Shirai and colleagues developed another SV-40 Tag targeted probasin promoter rodent model, this one in the Sprague Dawley rat [14]. However, this model has not been extensively characterized. Accordingly, we set out to thoroughly characterize the histopathology of autochthonous prostate cancer in the SV-40 Tag rat.

The goal of this study was to define appropriate windows of opportunity for pre-clinical trails using the SV-40 Tag rat. We report the incidence and pathological changes within the VP and DLP as a function of time throughout the natural history of prostate cancer in SV-40 Tag rats. Furthermore, we evaluated the role of sex steroid receptor and IGF-1 signaling proteins, cell proliferation, and apoptosis in the development of prostate cancer in this model. This study should aid in the rational design of chemoprevention, intervention, regression, and therapeutic studies using prostate cancer rodent models.

## Methods

### Animals

Animal care and use were conducted according to established guidelines approved by the National Institutes of Health and the Institutional Animal Care and Use Committee at the University of Alabama at Birmingham. Animals were housed in rooms maintained at 24 ± 1°C with a 12 hr light-dark cycle. All animals received powdered phytoestrogen-free AIN-76A diet (Harlan Teklad Global Diets, Wilmington, DE) and tap water.

SV-40 Tag rat breeders were provided for us by Dr. Tomoyuki Shirai of Nagoya City University Medical School *via *Drs. Gail Prins and Steve Swanson of the University of Illinois at Chicago. Heterozygous SV-40 Tag females were crossed with non-transgenic males to generate heterozygous SV-40 Tag male offspring (SV-40 SD females × SD male breeders). Day 21 post-conception females were anesthetized and the offspring were caesarian-derived to yield mycoplasma-free offspring that were used to establish our colony. At 3 weeks of age, the offspring were weaned and tails were clipped. DNA was extracted using a DNeasy Tissue Kit (Qiagen, Valencia, CA), and a PCR-based screening assay was performed to evaluate transgene incorporation [13-15].

Rats were necropsied starting at day 1 post-partum, once per week from 1 to 6 weeks, and every 2 weeks starting at 6 weeks of age and concluding at 40 weeks. At time of dissection, prostates were excised, weighed, and flash frozen in liquid nitrogen. Because it is unclear which lobe in the rodent resembles the human peripheral zone, the prostatic location in humans where prostate cancer normally occurs [16], we chose to analyze both the DLP, which has historically been referred to as the homologue of the human prostate [17] and the VP. Blood was collected at sacrifice, centrifuged at 3,000 × *g *and serum was stored at -80°C until time of analysis.

### Histopathology

At necropsy, organs were examined for gross abnormalities. Macro-metastasis to the bone, abdominal wall, lymph nodes, liver, kidney, and lung was investigated. Prostate, testes, seminal vesicles and tumor weights were also recorded. The prostate and organs of suspected metastasis were placed in cassettes, immersed in 10% formalin, dehydrated in a series of alcohol dilutions, fixed in xylene, embedded in paraffin wax, sliced into 5 μm sections, and placed on microscope slides as described by Folkvord *et al*. [18]. Sections were stained with hematoxylin and eosin prior to histopathological examination. Dr. Isam Eltoum, a Board Certified Pathologist, blindly scored each coded sample using the following grading scale developed specifically for rodents [16,19-21]: Grade 1 (non-cancerous), Grade 2 (low-grade PIN), Grade 3 (HG-PIN), Grade 4 (well-differentiated lesion), Grade 5 (moderately differentiated lesion), or Grade 6 (poorly differentiated lesion).

### Sex Steroid Hormone Concentrations in Blood Serum

Serum total testosterone, DHT, and estradiol concentrations were measured in the blood serum using coated-well enzyme- and radio-immunoassays (Diagnostic Systems Laboratories, Inc., Webster, TX) as described by the manufacturer. The following kits were used: DSL 10–4000 (total testosterone), DSL 9600 (DHT), and DSL 4800 (estradiol). All samples were run in duplicate with eight samples per group by Dr. Richard Parker (Obstetrics and Gynecology Department, UAB, Birmingham, AL). Standards provided by the manufacturer were used: 0.1–25 ng/mL for total testosterone, 25–2500 pg/mL for DHT, and 20–6000 pg/mL for estradiol. Sensitivity for total testosterone, DHT, and estradiol were 0.04 ng/mL, 4 pg/mL, and 7 pg/mL, respectively.

### IGF-1 Concentrations in Blood Serum

IGF-1 concentrations were measured in blood serum using a coated well ELISA kit as described by Diagnostic Systems Laboratories, Inc. All samples were run in duplicate with eight samples per group. Concentration (ng/mL) of samples and controls were determined by plotting the mean absorbance readings of the controls and unknowns against the mean absorbance readings of all standards in a four-parameter curve fit.

### Cell Proliferation

Prostate tissues (VP and DLP) were harvested and processed for detecting Ki-67, a marker of cell proliferation as previously described [21]. Slides were viewed using a Nikon Labophot-2 microscope (Nikon Corporation, Tokyo, Japan) and digitally recorded using a Nikon 8.0 Mega Pixels CoolPix 8700 Digital Camera (Nikon). For Ki67 quantitation, epithelial cells were counted using Image J software (Image J, NIH). The VP and DLP were analyzed separately (a minimum of 1,000 cells counted per lobe per slide. The epithelial cells staining positive (brown) for Ki67 were counted as well as the non-proliferative epithelial cells (stained blue). The proliferative index was defined as the number of positively stained epithelial cells divided by the total number of epithelial cells counted × 100. Twenty-eight week old TRAMP prostate tumor with and without Ki67 primary antibody was used as positive and negative controls, respectively.

### Apoptosis

The ApopTag^® ^Plus Peroxidase *In Situ *Apoptosis Detection Kit (Chemicon International, Temecula, CA) was used to measure apoptosis following the manufacturer's instructions. The apoptotic index was defined as the number of epithelial cells stained positive (brown) for apoptosis divided by the total number of epithelial cells counted × 100. Visualization was performed using a Nikon light microscope, Nikon digital camera, and analyzed using Image J software (NIH).

### Immunoblot Analyses

When possible, protein expression levels of sex steroid and growth factor receptors and their ligands were measured by western blot analysis as described previously [22]. Tissues (8 biological samples/treatment group) were homogenized in lysis buffer (1% Triton X-100, 10 mM Tris (pH 7.4), 1 mM EDTA, 1 mM EGTA, 1 mM Hepes (pH 7.6), 2 mM Na vanadate, 0.2 mM PMSF, 2 μg/ml leupeptin, 2 μg/ml aprotinin). Protein concentration of each sample was determined using the Pierce BCA Protein Assay (Pierce, Rockford, IL). The same quantity of protein from each sample was separated by SDS-PAGE and transferred to a nitrocellulose membrane (BioRad, Hercules, CA). The membranes were blocked and immunoblotted with appropriate antibodies purchased from Cell Signaling Technology (Danvers, MA): total Extracellular Regulating Kinases 1 and 2 (total-ERKs 1 and 2) (#9102) and Phosphatase and Tensin Homolog (PTEN) (#9554), and from Santa Cruz Biotechnology (Santa Cruz, CA): AR (SC-816), ER-alpha (SC-7207), insulin-like growth factor-1 receptor alpha (IGF-1R-α) (SC-712), phospho-Akt 1/2/3 (SC-7985), and β-actin (SC-47778) was used as a loading control. Positive protein controls purchased from the suppliers of the corresponding antibodies and the use of Kaleidoscope Precision Plus Protein and Pre-stained SDS-PAGE Broad Range standards (BioRad Hercules, CA) were employed to identify the protein of interest. After incubation with HRP-conjugated secondary antibody (Cell Signaling Technology), bands were detected using chemiluminescence (Pierce) and exposed to X-ray radiography film. Band intensity was quantified using scanning densitometry.

### Enzyme Linked Immunosorbent Assays

IGF-1 protein levels were quantified in the prostate by ELISA as described by Crowther *et al*. [23] with modifications [22]. Prior to analysis, kinetic curves were set up to establish zero order kinetics. Samples were run in triplicate and the absorbance at 450 nm was read in an OPTI max Microplate reader (Molecular Devices, Sunnyvale, CA). Rat liver with and without IGF-1 primary antibody was used as a positive and negative control, respectively.

### Statistics

Statistical comparisons were performed using two-tailed Students t-test assuming unequal variances for immunoblot analysis and ELISA. Trend analysis was implemented to compare cell proliferation and apoptosis between non-transgenic and SV-40 Tag rats. *P *< 0.05 was considered to be significant. GraphPad™Prism 5.0 and GraphPad™ InStat 3.0 (San Diego, CA) were used to assist in statistical analysis.

## Results

### Histopathology

*Via *the ontogeny study, we found that precursor lesion and prostate tumor progression was more rapid in the VP than the DLP of SV-40 Tag rats (Table 1). Prostates were graded normal (Grade 1) from day 1 through 1 week post-partum. However, low-grade PIN (Grade 2) quickly developed as early as 2 weeks of age in the VP and DLP. High-grade PIN (Grade 3), the precursor to prostate adenocarcinoma, developed as early as 4 and 5 to 6 weeks of age in the VP and DLP, respectively. None of the animals aged 8 weeks or younger developed cancerous lesions (Grade 4 or greater).

**Table 1:** Histopathological analysis of the ventral and dorsolateral prostate lobes of SV-40 Tag rats fed control AIN-76A diet

		Histopathological Grades	Histopathological Grades
Age	*n*	(Ventral Prostate)	(Dorsolateral prostate)

1 day	2	1, 1	1, 1

1 week	4	1, 1, 1, 1	1, 1, 1, 1

2 weeks	6	2, 2, 2, 2, 2, 2	1, 1, 2, 2, 2, 2

3 weeks	2	2, 2	2, 2

4 weeks	2	3, 3	2, 2

5 weeks	4	2, 2, 3, 3	1, 2, 2, 3

6 weeks	3	3, 3, 3	3, 3, 3

8 weeks	5	3, 3, 3, 3, 3	3, 3, 3, 3, 3

10 weeks	5	3, 3, 3, 4, 4	3, 3, 3, 3, 3

12 weeks	5	4, 4, 4, 4, 5	3, 3, 3, 3, 3

14 weeks	5	4, 4, 4, 4, 4	4, 4, 4, 4, 4

16 weeks	5	4, 5, 5, 5, 5	3, 3, 4, 4, 4

18 weeks	5	4, 5, 5, 5, 5	3, 4, 4, 4, 4

20 weeks	4	5, 5, 5, 5	4, 4, 4, 4

22 weeks	4	5, 5, 5, 5	5, 5, 5, 5

24 weeks	4	5, 5, 5, 5	5, 5, 5, 5

26 weeks	4	5, 5, 5, 6	3, 5, 6, 6

28 weeks	3	5, 6, 6	5, 5, 6

30 weeks	3	4, 6, 6	4, 6, 6

32 weeks	3	5, 5, 5	5, 5, 5

36 weeks	3	5, 5, 5	5, 5, 5

40 weeks	4	5, 5, 5, 5	5, 5, 5, 5

Tissue sections were evaluated for histopathological scores of 1 (no tumor), 2 (low grade PIN), 3 (high-grade PIN), 4 (well-differentiated tumor), 5 (moderately differentiated tumor), and 6 (poorly differentiated tumor) depending on the presence and progression of lesions. Individual grades are presented for each age group and lobe.

The first incidence of prostate cancer (well-differentiated lesions/Grade 4) developed at 10 and 14 weeks of age in the VP and DLP, respectively. By 10 weeks of age, 40% of animals developed prostate cancer in the VP, whereas by 14 weeks of age 100% of animals developed prostate cancer in the DLP. In addition, 100% of the rats had prostate cancer in each lobe by 14 weeks of age. Moderately-differentiated lesions (Grade 5) developed as early as 16 and 22 weeks of age in the VP and DLP, respectively. Poorly-differentiated lesions (Grade 6) developed at 26–30 weeks of age in the VP and DLP. When one lobe demonstrated Grade 6 lesions, the other lobe usually also exhibited Grade 6 lesions. Interestingly, from 32–40 weeks only moderately-differentiated lesions (Grade 5) and not poorly differentiated lesions (Grade 6) were found. Only 5% of SV-40 Tag rats developed macroscopic prostate tumors in which prostate lobes were indistinguishable at dissection (3.18 ± 2.16 grams). In 30 week old SV-40 Tag rats which developed Grade 4–6 lesions without macroscopic tumors, the prostate tissue weighed 1.27 ± 0.19 grams. This is to be compared to age matched non-transgenic rat prostates weighing 1.04 ± 0.20 grams. Metastasis to the pelvic lymph nodes, urethra, or kidneys was observed in less than 5% of SV-40 Tag rats at 30 weeks of age or older. Macro- or micro-metastasis was not observed in any animals younger than 30 weeks of age.

### Blood Sex Steroid Concentrations

**Figure 1:**
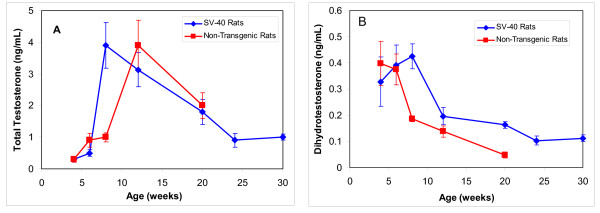
**Androgen concentrations in the blood serum of SV-40 Tag and non-transgenic rats fed AIN-76A diet**. A) Testosterone concentrations. B) Dihydrotestosterone concentrations. Each age group had at least 5 animals per group. Values are presented as the mean ± SEM. In non-transgenic rats, androgen concentrations were not determined at 24 and 30 weeks.

Circulating androgens were measured in the blood *via *immunoassay to investigate their potential role in prostate cancer etiology. In non-transgenic rats peak total testosterone concentrations were found at 12 weeks (Figure 1A). However, in SV-40 Tag rats, peak testosterone concentrations were found at 8 weeks at concentrations that were 4 fold of those in non-transgenic rats a the same age (p < 0.001). This preceded prostate cancer development in the VP by 2 weeks in SV-40 Tag rats (Table 1). Total testosterone in the blood serum of SV-40 Tag rats at 8, 12, 20, and 30 weeks of age were significantly higher by 11-fold, 10-fold, 6-fold, and 3.3-fold, respectively, when compared to concentrations at 4 weeks of age in SV-40 Tag rats (*P *< 0.05).

In non-transgenic rats, serum DHT concentrations were highest in the prepubertal period, that is, at 4–6 weeks old (Figure 1B). Thereafter, DHT concentrations decreased. In SV-40 Tag rats, serum DHT concentrations were similar at 4–6 weeks, but at 8 weeks, DHT concentrations were found to be significantly increased 2.3-fold in the SV-40 Tag rats when compared to non-transgenic rats at the same age (p < 0.001). Then in SV-40 Tag rats, DHT concentrations progressively decreased. Increasing DHT concentrations at 8 weeks in SV-40 Tag rats accompanied PIN development between 4 to 8 weeks of age (Figure 1B and Table 1). Calculating DHT to testosterone ratios revealed significantly higher values at 4 weeks of age when compared to 8 weeks or older in SV-40 Tag rats and non-transgenic rats (data not shown). At all ages investigated, however, there were no significant differences in DHT to testosterone ratios between transgenic and non-transgenic rats.

**Figure 2:**
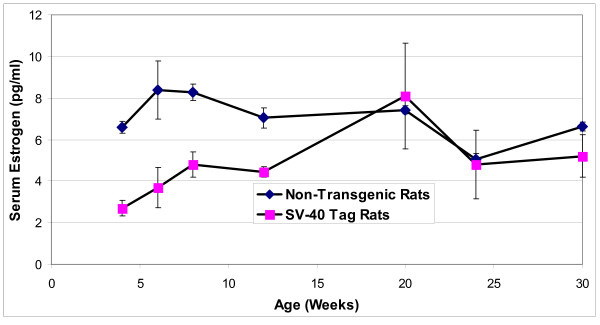
**Estrogen concentrations in the blood serum of SV-40 Tag and non-transgenic rats fed AIN-76A diet**. Each age group had at least 5 animals per group. Values are presented as the mean ± SEM.

Serum estradiol 17-β concentrations in non-transgenic rats were highest at 6 to 8 weeks, and then gradually decreased (Figure 2). In the SV-40 Tag rats, serum estradiol 17-β concentrations at 4, 6, 8 and 12 weeks were significantly lower than those in non-transgenic rats at these same ages (*P *< 0.05). Thereafter, serum estradiol 17-β from SV-40 Tag rats increased to concentrations similar to those of non-transgenic rats at 20 to 30 weeks. In non-transgenic compared to SV-40 Tag rats, estrogen to testosterone ratios were calculated to be 24 and 5.5 fold higher at 4 and 8 weeks, respectively. At other times that were investigated, the estrogen to testosterone ratios were not significantly different. From 4 weeks through 20 weeks, the estrogen to DHT ratios were 2–4 fold higher in non-transgenic compared to SV-40 Tag rats.

### IGF-1 Concentrations in Blood Serum

**Figure 3:**
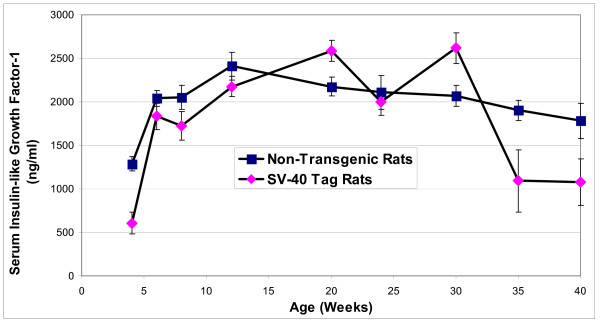
**Insulin-like Growth Factor-1 concentrations in the blood serum of SV-40 Tag and non-transgenic rats fed AIN-76A diet**. Each age group had at least 5 animals per group. Values are presented as the mean ± SEM.

IGF-1 concentrations in the blood of non-transgenic and SV-40 Tag rats were found to be lowest at the youngest age investigated, 4 weeks postpartum (Figure 3). In non-transgenic rats, IGF-1 concentrations increased 2-fold and peaked at 12 weeks, and then gradually decreased with age. In the SV-40 Tag rats, serum IGF-1 concentrations significantly increased by 3-fold between 4 and 6 weeks of age (*P *< 0.05), increased steadily thereafter, and peaked at 20 to 30 weeks of age. Thereafter, serum IGF-1 concentrations significantly decreased by 2.5 fold at 36–40 weeks of peak concentrations. Only at 4 weeks postpartum were serum IGF-1 concentrations found to be significantly different between SV-40 Tag and non-transgenic rats with the concentrations being two fold less in the SV-40 Tag rats (P < 0.05).

### Cell Proliferation and Apoptosis

**Figure 4:**
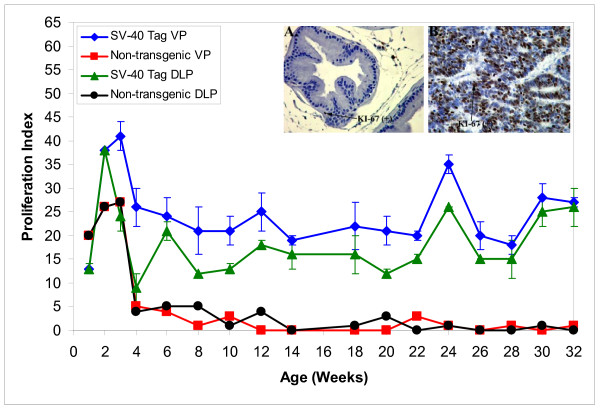
**Cell proliferation as determined by immunohistochemical staining of Ki-67 in the ventral and dorsolateral prostates of SV-40 Tag and non-transgenic rats fed AIN-76A diet**. Each age group had at least 3 transgenic and 2 non-transgenic animals per group. Values are presented as total number of proliferating epithelial cells divided by total number of epithelial cells × 100 ± SEM. Picture inserts are examples of brown staining for Ki-67 in ventral prostates of 32 week old rats. A) Example of rare proliferating cells (brown staining – arrow) for KI-67 in ventral prostate from 12 week old non-transgenic rat. B) Numerous proliferating Ki-67 positive cells (brown stain) in ventral prostate of a SV-40 Tag rat; a prostatic duct with high grade intra-epithelial lesion showing marked tufting, papillary formation, nuclear crowding and high nuclear cytoplasmic ratio.

Cell proliferation and apoptosis were investigated at different ages to gain insight into critical stages of prostate cancer development. Trend analysis demonstrated that starting at 2 weeks postpartum, cell proliferative indices were significantly higher in the VP and DLP of SV-40 Tag rats than in non-transgenic rats (*P *< 0.05) (Figure 4). At 10 weeks of age, when 100% of the rats had developed high grade-PIN, cell proliferation was 7-fold and 13 fold higher in the VP and DLP of SV-40 Tag rats than in those of non-transgenic rats. Furthermore, at 12 weeks of age, the cell proliferative indices in the VP and DLP of SV-40 Tag rats were 50 fold and 4.5 fold higher than those in non-transgenic rats. The increase in cell proliferation in SV-40 Tag versus non-transgenic rat prostate continued through the time of necropsy at 30–32 weeks where the proliferation indices were 54 and 51 fold higher in the VP and DLP, respectively. The picture inserts in Figure 4 illustrate typical staining for the Ki-67 protein in the VP of 12 week old SV-40 Tag and non-transgenic rats.

**Figure 5:**
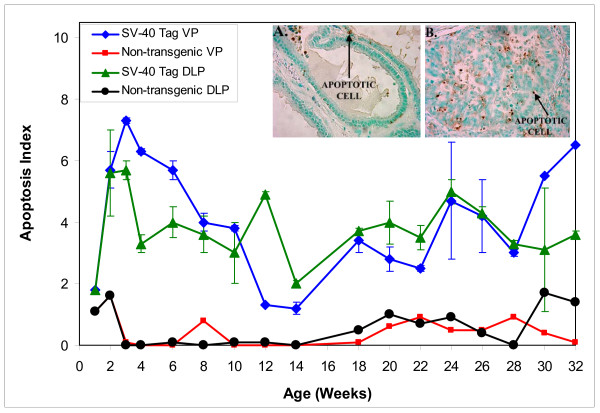
**Apoptosis in the ventral and dorsolateral prostates of SV-40 Tag and non-transgenic rats fed control AIN-76A diet, starting at day one**. Each age group had at least 3 transgenic and 2 non-transgenic animals per group. Values are presented as total number of apoptotic epithelial cells divided by total number of epithelial cells × 100 ± SEM. Picture inserts are examples of brown staining for apoptotic cells in ventral prostates of 32 week old A) non-transgenic rats and B) SV-40 Tag rats. In A: a normal prostatic duct lined by cuboidal-to-columnar epithelial cells that have abundant cytoplasm and uniform non-overlapping nuclei (green staining). There are infrequent apoptotic cells (brown staining – arrow). In B: numerous apoptotic bodies with solid mass of tumor, minimal glandular formation, nuclear crowding and cells with high nuclear cytoplasmic ratio.

At all ages investigated, apoptotic indices were higher in VP and DLP of SV-40 rats than in non-transgenic rats. At 10 weeks of age, apoptotic index in the VP of SV-40 Tag rats was 3.8% compared to less than 0.01% in the VP of non-transgenic rats (Figure 5). The lowest apoptotic indices in the VP of SV-40 Tag rats were observed between 12–14 weeks of age, the stretch of time in which 100% of animals develop prostate cancer. The picture inserts illustrate typical staining for apoptotic cells in the VP of 12 week old SV-40 Tag rats and non-transgenic rats. Similarly, the lowest apoptotic indices in the DLP of SV-40 Tag rats occurred at 14 weeks of age, at the time that prostate cancer developed in the DLP.

### Biomarker Regulation

After observing high rate of cell proliferation coupled with low apoptosis, specifically at 12–14 weeks of age when compared to the proliferative and apoptotic indices at younger and older ages in the VP of SV-40 Tag rats, we investigated protein biomarkers. AR protein expression in the VP, but not the DLP, of SV-40 Tag rats was significantly increased by 85% when compared to those in non-transgenic rats (Table 2). In the VP and DLP of 12 week old SV-40 Tag rats, we found that protein expression of ER-alpha was significantly down-regulated by approximately 50% compared to the VP and DLP of non-transgenic rats. IGF-1 protein expressions in the VP and DLP of SV-40 Tag rats were significantly increased when compared to non-transgenic rats by 10% and 46%, respectively. Moreover, IGF-1R protein expression in the VP and DLP were significantly up-regulated by 153% and 38% in SV-40 Tag rats compared to non-transgenic rats. In addition, total-ERKs 1 and 2 protein expressions in the VP of SV-40 Tag rats were significantly increased by 3-fold. Likewise, total-ERKs 1 and 2 protein expressions in the DLP were significantly up-regulated by 1.7-fold and 1.6-fold, respectively when compared to those in non-transgenic rats.

**Table 2:** Biomarker protein expressions in the prostate lobes of 12 week old non-transgenic and SV-40 Tag rats

	Non-transgenic Ventral Prostate	SV-40 Tag Ventral Prostate	Non-transgenic Dorsolateral Prostate	SV-40 Tag Dorsolateral Prostate
Androgen Receptor	100 ± 12	185 ± 22*	100 ± 7	116 ± 11

Estrogen Receptor-alpha	100 ± 6	49 ± 7*	100 ± 9	51 ± 7*

Insulin-like Growth Factor-1	100 ± 3	110 ± 4*	100 ± 2	146 ± 6*

Insulin-like Growth Factor-1-alpha Receptor	100 ± 3	253 ± 32*	100 ± 6	138 ± 3*

Total-Extracellular Regulating Kinase-1	100 ± 5	302 ± 64*	100 ± 19	174 ± 21*

Total-Extracellular Regulating Kinase-2	100 ± 4	298 ± 58**	100 ± 16	157 ± 16*

Protein expressions were determined by immunoblot analysis, except for IGF-1 levels that were determined by ELISA (*n *= 6 per group). Protein expressions for non-transgenic rats were set to 100 and those of transgenic rats are presented as percent of non-transgenic rats ± SEM. *P < 0.05 and **P < 0.01 compared to non-transgenic rats by two-tailed Students t test.

## Discussion

The detailed ontogeny study revealed that prostate cancer in SV-40 Tag rats developed first and most extensively in the VP as contrasted to the TRAMP (mouse) model where prostate cancer is reported to originate in the DP/DLP [20,24]. From our ontogeny study in SV-40 Tag rats, we found that low grade PIN (Grade 2) developed as early as two weeks in the VP and DLP. This is 2 weeks earlier than had been previously reported [14]. High-grade PIN (Grade 3), the precursor to prostate adenocarcinoma, was observed as early as 4 weeks in the VP and at 5 to 6 weeks in the DLP. The first incidence of prostate cancer (well-differentiated lesions – Grade 4) developed at 10 and 14 weeks of age in the VP and DLP, respectively. By 12 weeks of age 100% of SV-40 Tag rats developed prostate cancer in the VP, whereas it was not until 14 weeks of age that 100% animals developed prostate cancer (Grade 4) in the DLP. Hence, this detailed ontogeny study revealed that prostate cancer occurred 3–5 weeks earlier than at 15 weeks of age as previously reported for SV-40 Tag rats [25]. In contrast, PIN and invasive adenocarcinoma developed in TRAMP mice at 10 and 18 weeks of age, respectively [26]. Even though SV-40 Tag rats are larger than TRAMP mice (526 grams and 36 grams, respectively at 30 weeks), prostate tumors are larger in TRAMP mice (7.59 ± 0.1.49 grams) when compared to prostate tumors/lesions in SV-40 Tag rats (1.71 ± 0.37 grams). Furthermore, when one considers prostate weights in non-transgenic mice (83 ± 9 mg) and non-transgenic rats (1.04 ± 0.20 gram) at 30 weeks, this results in prostate tumor to prostate tissue weight increases of 91 fold in mice and 1.6 fold in rats. The differences in prostate cancer origin, progression and tumor size in these models illustrates the fact that despite both being driven by the probasin/SV-40 Tag transgene, there are stark differences in the development of prostate cancer in these two rodent model.

Based on the ontogeny study, we chose 12 weeks of age as time at which to kill rats for future mechanism of action studies since these animals were transitioning from high grade-PIN to prostate adenocarcinoma. For future chemoprevention studies, we suggest 30 weeks of age to sacrifice rats since the majority of animals at this age have developed poorly differentiated tumors, but not severe morbidity. Animals aged 32–40 weeks were not observed with poorly differentiated tumors, most likely due to the fact that several animals over the age of 30 weeks died because of disease progression and related factors prior to their designated sacrifice date.

Testosterone concentrations were lowest in the youngest animals investigated (4 weeks old), peaked at 8 and 12 weeks in SV-40 Tag and non-transgenic rats, respectively, and declined thereafter to level off at 24 to 30 weeks of age. DHT concentrations were moderately high at 4 weeks of age, peaked at 8 weeks in SV-40 Tag rats, and decreased thereafter, with the lowest concentrations at 24 to 30 weeks. Comparing blood androgen levels at 8 weeks, we found the testosterone and DHT concentrations to be 3.9-fold and 2.3-fold higher in SV-40 Tag rats than in non-transgenic rats. Since the androgen levels peak at 8 weeks, it is quiet plausible that increasing high concentrations of these androgens in the SV-40 Tag rats at time of high grade-PIN facilitates early prostate cancer development (well differentiated lesions). These changes may lay the foundation and provide the optimal environment for further prostate cancer progression.

As reviewed by Prins *et al*., researchers have speculated that estrogens may play a role in prostate cancer causation and promotion [27]. Our assay of estradiol-17β in blood showed that at 4–12 weeks, non-transgenic rats compared to SV-40 Tag rats had two fold more serum estradiol. Although the estrogen to DHT ratio, but not the estrogen to testosterone ratio, increased in non-transgenic rats and SV-40 Tag rats as a function of age, this ratio was constantly higher in non-transgenic rats compared to SV-40 Tag rats. Therefore, the estrogen to androgen ratio was not correlated with prostate cancer development in the SV-40 Tag rats compared to non-transgenic rats. Testosterone concentrations (free and conjugated testosterone) in the blood serum were consistent with previously reported levels in SV-40 Tag rats [28-30], whereas DHT and estradiol concentrations in SV-40 Tag rats have yet to be published. Moreover, testosterone and DHT concentrations, as well as DHT to testosterone ratios in the blood serum of Sprague Dawley non-transgenic rats, were within range of previously reported values [31-33].

Elevated levels of blood IGF-1 have been associated with an increased risk of advanced stage prostate cancer [7,34-36]. Contrary to what might be expected, serum IGF-1 levels were not the highest later in life during the progression to poorly differentiated (advanced) prostate cancer in the SV-40 Tag rats. Despite a poorly differentiated tumor frequency of 66% at 30 weeks of age, serum IGF-1 concentrations gradually declined after 30 weeks of age. The lowest post-pubertal levels were noted in the oldest animals (40 weeks of age). IGF-1 levels in the blood serum of SV-40 Tag rats as they aged mimicked the pattern observed in non-transgenic rats and humans but at higher concentrations. This demonstrates that IGF-1 levels in the blood serum may not be the optimal marker for cancer progression in this model, but simply reflects IGF-1 production from the liver [37].

Because high levels of sex steroids and growth factors can often lead to changes in cell turnover, we measured cell proliferation and apoptosis. Cell proliferation in non-transgenic rats was similar in the VP and DLP. Likewise, in SV-40 Tag rats, cell proliferation was similar in the VP and DLP, but cell proliferation was significantly higher in prostates of SV-40 Tag rats (1.5- to 54-fold higher, the latter in adult animals). Specifically, proliferation was significantly higher in the VP at an earlier age (2 weeks postpartum) than in the DLP (6 weeks). Increased cell proliferation is consistent with high grade-PIN and eventually cancer lesions developing earlier in the VP than in the DLP.

Apoptotic indices were similar in the VP and DLP of non-transgenic rats and in the VP and DLP of SV-40 Tag rats. However, there was a significantly higher rate of apoptosis in prostates of SV-40 Tag rats compared to non-transgenic rats. We speculate that the increase in apoptosis occurs as a consequence of increased cell proliferation and DNA damaged cells being less stable and more prone to cell death. The onset of apoptosis followed the initial surge in cell proliferation and concomitant with low grade PIN development in both the VP and DLP. Most striking is the dramatic decreases in apoptosis from 10–14 weeks in the VP and from 12 to 18 weeks in the DLP. This mirrors the transition from high grade-PIN to well differentiated lesions (cancer). Hence, the increase in cell proliferation and decrease in apoptosis correlate very well with cancer formation in SV-40 Tag rats.

After noting changes in cell proliferation and apoptosis between SV-40 Tag and non-transgenic rats, we investigated proteins that could serve as biomarkers of action. AR protein expression in the VP was higher in 12 week old SV-40 Tag rats when compared to non-transgenic rats. Coupling up-regulated AR with the fact that testosterone and DHT concentrations were higher in SV-40 Tag rats makes a strong case for increased cell proliferation in the prostate and of prostate tumor development in this animal model. Also, we found ER-alpha protein expression was decreased in both the VP and DLP of SV-40 Tag rats. Concomitantly, we found significantly lower serum estrogen concentrations in SV-40 Tag rats at 4–12 weeks. These lower serum estrogen concentrations can be interrupted as reduced potential for estrogen action in SV-40 Tag prostate and perhaps reduced anti-androgenic action.

IGF-1 levels in the VP and DLP were elevated in SV-40 Tag rats compared to those in non-transgenic rats. Therefore, IGF-1 in the prostate tissue appears to be an important protein involved in prostate cancer development in this model. In addition to the growth factor ligand IGF-1 being altered, IGF-1R and total-ERKs 1 and 2 protein expressions were up-regulated in the VP when SV-40 Tag rats were compared to non-transgenic rats. Taken together, the up-regulation of the AR, IGF-1 signaling proteins, and their downstream effectors (ERKs) suggest a potential mechanism for prostate cancer development in the SV-40 Tag rat model. Therefore, chemopreventive and therapeutic agents with the ability to modulate these pathways make attractive candidates for prevention and therapy in this model.

The SV-40 Tag rat and TRAMP models are similar in some respects. As observed in humans, prostate cancer in SV-40 rats is initiated early in life [14,38]. In both models, the SV-40 large T antigen is under control of the probasin promoter allowing androgen-regulated protein expression specific to the epithelium of the prostate. Transformation in the prostate occurs when the SV-40 large T antigen acts as an oncoprotein *via *interactions with retinoblastoma [39] and p53 tumor suppressor gene products [40,41]. The small t antigen may also play a role in promotion by interacting with protein phosphatases [42].

## Conclusion

The SV-40 Tag rat provides an alternative transgenic rodent model of prostate cancer. Our ontogeny study demonstrates that transition from PIN to well differentiated tumor formation occurs from 10 to 12 weeks in the VP and at 14 weeks in the DLP of SV-40 Tag rats. The development of prostate cancer first in the VP of SV-40 rats is to be contrasted to that in TRAMP mice where it first develops in the DLP. An advantage of the SV-40 Tag rat over the TRAMP model is that prostates at 12 weeks in the former are 25 times as large (781 ± 50 mg) as compared in the latter (31 ± 2 mg), allowing for a greater number of mechanism of action experiments to be conducted using fewer animals. Although the cost of housing rats is roughly twice as expensive as mice, the ability to use fewer animals reduces the overall cost in the long run.

The SV-40 Tag rat displays other unique characteristics including reported complete androgen dependence, low metastasis, and androgen-independent metastasizing taste bud neuroblastomas [43]. In fact, Asamoto *et al*. reported that castration at 20 weeks of age (after prostate tumors had developed) caused complete regression and involution of these adenocarcinomas and earlier castration at 5 weeks of age completely inhibited prostate adenocarcinomas [14]. More detailed studies are merited to confirm this important characteristic. In contrast, castrated 12 week old TRAMP mice still developed poorly differentiated tumors with metastatic potential [15,44]. When castrated at 4 weeks of age (prior to high grade-PIN development), some TRAMP mice developed androgen-independent prostate cancer. Although the SV-40 Tag rat may never replace the TRAMP model, it is an additional model that can be tailored to specific studies. In the TRAMP model, the ability to progress to androgen independence [15,44,45], allows it to be used to study late stage prostate cancer. On the other hand, because of its reported androgen dependence [14] and the fact that most human prostate cancer is androgen dependent before androgen ablation treatment, the SV-40 Tag rat model could be useful in studying early stage prostate cancer. The models compliment each other. It is currently unknown which lobe in the rat prostate is homologous to the peripheral zone, where prostate cancer occurs in the humans. Therefore, both the DLP and VP should be investigated. Since prostate cancer develops first and to the greatest degree in the DP/DLP of the TRAMP model, it is an ideal model to study prostate cancer in that portion of the prostate. On the other hand, prostate cancer develops first and most extensively in the VP prostate of SV-40 Tag rats, thus providing a basis to study prostate cancer in the VP with this model.

## Abbreviations

AR: androgen receptor; DHT: dihydrotestosterone; DLP: dorsolateral prostate; ELISA: enyme-linked immunosorbent assay; ERK: extracellular regulating kinase; ER-alpha: Estrogen receptor-alpha; IGF: insulin growth factor; IGF-1R: IGF-1 receptor; MAPK: mitogen-activated protein kinase; PIN: prostatic intraepithelial neoplasia; SV-40 Tag: Simian Virus large T antigen; TRAMP: Transgenic Adenocarcinoma Mouse Prostate; VP: ventral prostate.

## Competing interests

The authors declare that they have no competing interests.

## Authors' contributions

CEH, LMC, BJP and JW carried out the ontogeny study, cell proliferation and apoptosis assays, and analysis of protein expression for mechanism of action. IAE analyzed and interpreted the pathology data. CH drafted the manuscript and LMC, BJP and JW assisted in writing the manuscript. TS provided permission to use the SV-40 Tag model and CAL proposed the study design and assisted in writing the manuscript. All authors read and approved the final manuscript.

## Pre-publication history

The pre-publication history for this paper can be accessed here:

http://www.biomedcentral.com/1471-2407/9/30/prepub
